# Frequency-selective propagation of localized spoof surface plasmons in a graded plasmonic resonator chain

**DOI:** 10.1038/srep25576

**Published:** 2016-05-05

**Authors:** Zhen Gao, Fei Gao, Kunal Krishnaraj Shastri, Baile Zhang

**Affiliations:** 1 Division of Physics and Applied Physics, School of Physical and Mathematical Sciences, Nanyang Technological University, Singapore 637371, Singapore; 2 Centre for Disruptive Photonic Technologies, Nanyang Technological University, Singapore 637371, Singapore

## Abstract

Localized spoof surface plasmon polaritons (spoof-SPPs) in a graded spoof-plasmonic resonator chain with linearly increasing spacing are experimentally investigated at microwave frequencies. Transmission measurements and direct near-field mappings on this graded chain show that the propagation of localized spoof-SPPs can be cutoff at different positions along the graded chain under different frequencies due to the graded coupling between adjacent resonators. This mechanism can be used to guide localized spoof-SPPs in the graded chain to specific positions depending on the frequency and thereby implement a device that can work as a selective switch in integrated plasmonic circuits.

It is well-established that structured metallic surfaces support unique electromagnetic surface modes at subwavelength scales ranging from microwave to far-infrared^1^,^2^,^3^. These surface modes are widely termed as “designer” or “spoof” surface plasmon polaritons (spoof-SPPs) since they have similar characteristics to surface plasmon polaritons (SPPs) found in metal-dielectric interfaces at visible frequencies. Owning to their tunability, spoof-SPPs have many exciting technological prospects, particularly in subwavelength manipulation of electromagnetic waves^4^,^5^,^6^,^7^,^8^,^9^,^10^,^11^,^12^. Phenomena such as the slow-light “rainbow” effect which involves stopping propagating spoof-SPPs on graded metallic structures at different positions for different frequencies have been demonstrated in the last decade^13^,^14^. Similar effects have also been verified in both visible^15^ and microwave^16^,^17^ regimes.

While most of these previous studies focused on propagating spoof-SPPs, recently, localized spoof-SPPs have been demonstrated using spoof-plasmonic resonators^18^,^19^,^20^,^21^,^22^,^23^. In this report, we propose and experimentally demonstrate a one-dimensional adiabatically graded spoof-plasmonic resonator chain that can transport localized spoof-SPPs to different positions at different frequencies. This resonator chain consists of a graded coupled-resonator optical waveguide (CROW)^24^ with the spacing between resonators increasing linearly along the chain. This structure can work as a selective switch and can have potential applications in integrated plasmonic circuits.

## Results

**Figure 1:**
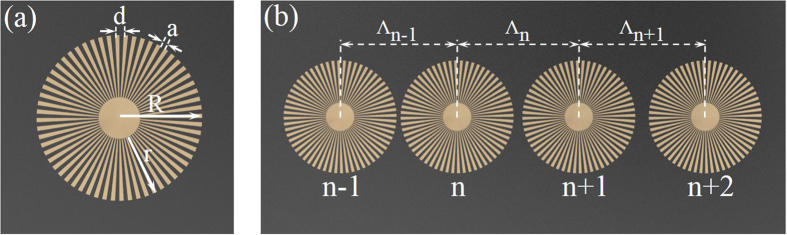
Schematic of single spoof-plasmonic resonator and graded spoof-plasmonic resonator chain. (**a**) Schematic picture (top view) of the spoof-plasmonic resonator with radius R = 12 mm. The depth, width and periodicity of grooves are r = 9 mm, a = 0.625 mm and d = 1.255 mm, respectively. (**b**) Schematic of the graded spoof-plasmonic resonator chain that consists of resonators with linearly increased spacing.

In this experiment we use a recently proposed spoof-plasmonic resonator^19^ (shown in Fig. 1a) as the fundamental building block of the graded chain. The resonator consists of an ultrathin corrugated metallic disk of copper of thickness 0.018 mm printed on a dielectric substrate (Rogers RT 5880) of thickness 0.254 mm using standard printed circuit board (PCB) technology. A graded change of coupling between the resonators is induced by linearly increasing the spacing between neighboring resonators along the chain. In our study we use a chain with eleven spoof-plasmonic resonators, and the spacing between adjacent resonators obeys the following rule: Λ_n_ = 25 + (n−1) mm, where Λ_n_ denotes the spacing between the n-th and (n + 1)-th resonators, as shown in Fig. 1b (top view).

**Figure 2:**
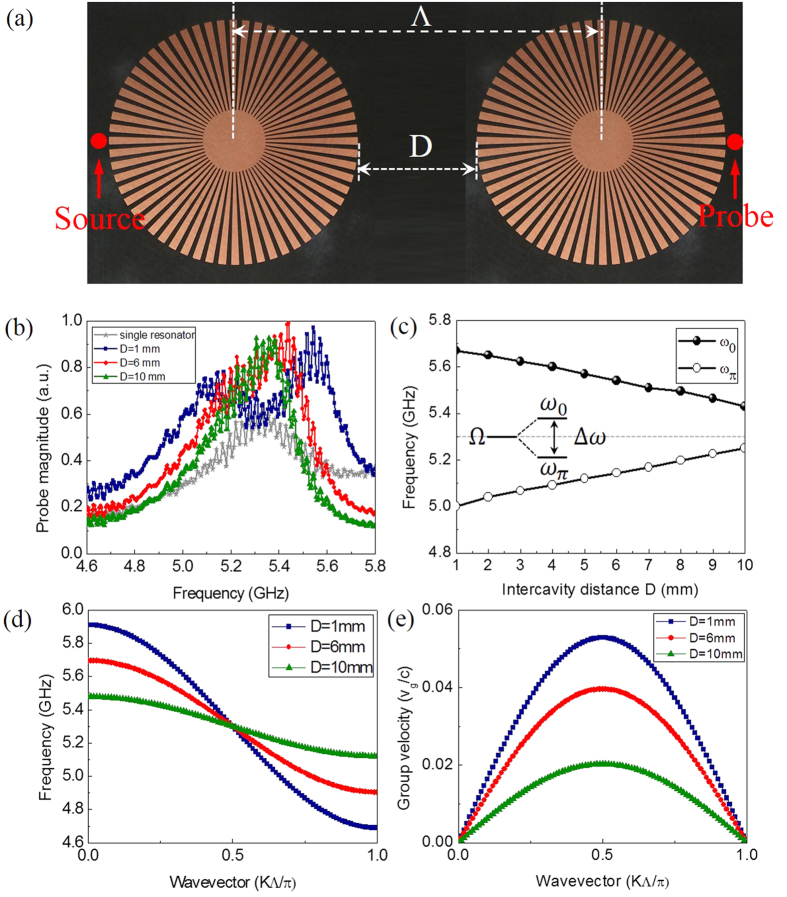
Coupling between two spoof-plasmonic resonators. (**a**) Picture of two coupled spoof-plasmonic resonators with inter-cavity distance *D*. Red dots indicate monopole point source and probe. (**b**) The measured near-field transmission spectra of the quadrupole mode through a coupled-resonator dimer for different inter-cavity distances *D*. The near-field response spectrum of a single resonator (grey line) is plotted for comparison. (**c**) The dependence of the split resonance frequencies (*ω*_0_ and *ω*_*π*_) on the inter-cavity distance *D* between the two coupled resonators. Inset is a diagram showing the mode splitting of the resonator-dimer. (**d**) Dispersion curves calculated from experimentally retrieved coupling factors for the quadrupole modes with different inter-cavity distances *D*. (**e**) Group velocity curves calculated from experimentally retrieved coupling factors for the quadrupole modes with different inter-cavity distances *D*.

Since the characteristics of the spoof-SPP resonator chain mainly depend on the graded couplings between neighboring resonators, we first consider coupling between two resonators by exciting the quadrupole mode. To measure the near-field transmission spectra, two microwave monopole antennas, acting as source and probe respectively, are employed at the location of the two red dots close to the spoof-plasmonic resonators shown in Fig. 2a. The measured near-field transmission spectra through this coupled-resonator dimer for different inter-cavity distances *D* = 1 mm (blue line), *D* = 6 mm (red line) and *D* = 10 mm (green line) are presented in Fig. 2b. For comparison, the transmission spectrum for a single resonator (grey line) is also measured by placing the source and probe at opposite sides of an individual resonator. The resonance peak at 5.34 GHz observed in this case, corresponds to the quadrupole mode of a single spoof-SPP resonator. When the coupled-resonator dimer is excited with a monopole antenna, the quadrupole mode of an individual resonator at *Q* = 5.34 GHz is observed to split into two supermodes: at *ω*_π_ = 5.00 GHz and *ω*_0_ = 5.67 GHz for inter-cavity distance *D* = 1 mm, *ω*_*π*_ = 5.19 GHz and *ω*_0_ = 5.49 GHz for inter-cavity distance *D* = 6 mm, and *ω*_*π*_ = 5.25 GHz and *ω*_0_ = 5.42 GHz for inter-cavity distance *D* = 10 mm. Here the subscripts “π” and “0” respectively denote out-of-phase and in-phase phase relations for the two resonators after mode splitting. It is seen that decreasing the inter-cavity distance *D* increases the separation between two split resonances (which is a direct indication of the coupling strength), as expected from the tight-binding formalism that two cavities couple through the overlap of their evanescent modal tails^25^. Subsequently, the spectral position of the resonance peaks of the split quadrupole modes with variable inter-cavity distances *D* from 1 mm to 10 mm are measured and presented in Fig. 2c. It is apparent that the resonance frequencies of two split resonant modes approach the resonance frequency of the quadrupole mode of a single resonator (grey dashed line) as the inter-cavity distance *D* is increased.

Using an optical analogue of tight-binding approximation^25^, we can experimentally retrieve the coupling factor *κ* from the measured split resonance frequencies and the phase relation between the two coupled resonators. We obtain *κ*_1_ = 0.125 for inter-cavity distance *D* = 1 mm, *κ*_6_ = 0.056 for inter-cavity distance *D* = 6 mm, and *κ*_10_ = 0.0318 for inter-cavity distance *D* = 10 mm, respectively. It is apparent that a larger inter-cavity distance has a smaller coupling factor due to weaker coupling.

Next, we analyze the dispersion curves of a one-dimensional (1D) infinite spoof-plasmonic resonator chain with a constant inter-cavity distance. When the number of resonators is increased, a waveguiding band is expected due to the coupling of individual resonant modes^24^. The dispersion relations for the quadrupole mode can be calculated for different inter-cavity distances using Yariv’s formula: ω_K_ = Ω[1 + *κ*cos(*K*Λ)], where Ω is the resonant frequency of an individual resonator, *κ* is the inter-resonator coupling strength extracted from the mode splitting of two coupled resonators, *K* is the Bloch wavevector, and Λ is the periodicity of the coupled-resonator waveguide. The dispersion relations for inter-cavity distance *D* = 1 mm (blue line), *D* = 6 mm (red line), and *D* = 10 mm (green line) are presented in Fig. 2d. The waveguiding bandwidth is observed to get narrower as the inter-cavity distance gets larger since the increased distance induces the smaller coupling strength *κ*. In Fig. 2e we plot the normalized group velocity: 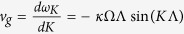 of the waveguiding band corresponding to waveguides with different inter-cavity distances *D* as a function of wave vector *K*. It can be seen that the group velocity has a maximum value at the coupled-cavity waveguiding band center, and decreases significantly as the band edges are approached.

**Figure 3:**
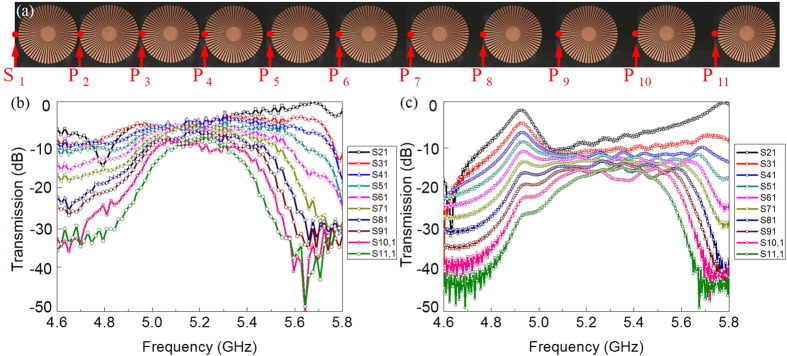
Measured and simulated transmission spectra of graded spoof-plasmonic resonator chain. (**a**) Picture (top view) of the fabricated graded spoof-plasmonic resonator chain with linearly increasing spacing. Positions of the source (S_1_) and probe (P_2_-P_11_) are indicated as red dots. (**b**) The measured transmission spectra through the graded spoof-plasmonic resonator chain for different probe positions. (**c**) The numerically simulated transmission spectra through the graded spoof-plasmonic resonator chain for different probe positions.

Finally, we consider a graded spoof-SPP resonator chain with distance between the resonators linearly increasing from the left-hand side (*D* = 1 mm) to the right-hand side (*D* = 10 mm) in steps of 1 mm (Fig. 3a). It should be noted that one cannot define the wavevector in a rigorous sense in a graded system due to the absence of translational invariance. However, if we assume each pair of neighboring spoof-SPP resonators as periodic chains with constant spacing, the dispersion relations are expected to change gradually along the chain as the spacing is linearly increased. Thus, the dispersion relations will vary as a function of position along the chain, roughly changing from the dispersion relation shown for *D* = 1 mm to that for *D* = 10 mm in Fig. 2d. The first pair of the spoof-SPP resonators (*D* = 1 mm) can be considered as a gate to determine the largest frequency range of the waveguiding band. With increasing spacing along the graded chain, the coupling strength between neighboring resonators decreases and the bandwidths as well as the cutoff frequencies reduce. At the end of the graded chain, only localized spoof-SPPs of the periodic chain with inter-cavity distance *D* = 10 mm (corresponding the last pair of resonators) can propagate through the chain. Therefore, the graded spoof-SPP resonator chain works like a filter to gradually get rid of the localized spoof-SPPs with frequency within the band region of *D* = 1 mm and outside the band region of *D* = 10 mm along the chain.

**Figure 4:**
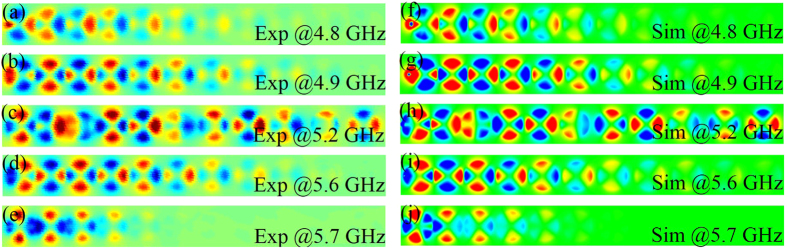
Measured and simulated electric near-field distributions. (**a**–**e**) The measured E_z_-field distribution 1 mm above the top of the graded spoof-plasmonic resonator chain at different frequencies. (**f**–**j**) The numerically simulated E_z_-field distribution 1 mm above the top of the graded spoof-plasmonic resonator chain at different frequencies.

We now discuss the experimental setup used to characterize the fabricated graded spoof-SPP resonator chain described above. We start by exiting the graded chain with a monopole antenna placed 1 mm away from the left-side of the first resonator (red dot S_1_ in Fig. 3a), and measure the transmission spectra of the localized spoof-SPPs with another monopole antenna acting as a probe. This probe is placed 1 mm above the graded chain and can freely move to measure both the amplitude and phase of the received signal at points indicated by red dots P_2_-P_11_ in Fig. 3a. Both monopole antennas are connected to a vector network analyzer (R&S ZVL-13) to transmit and receive the signal. We conduct the experiment in two steps to demonstrate the predictions made in the previous paragraph. First, we measure and simulate the transmission spectra at different positions (P_2_-P_11_) along the resonator chain to verify the graded transmission bandwidths. The measured and simulated results of the S parameters (transmission coefficients S21) are shown in Fig. 3b,c. We observe that the transmission bandwidths gradually decrease as the probe moves along the graded chain direction and is, hence, consistent with the dispersion curves in Fig. 2b. The transmission coefficients illustrated in Fig. 3b,c clearly indicate the highly frequency-selective property of the proposed structure. Compared to existing frequency-selective devices based on propagating spoof-SPPs^26^, the filtering performance of our structure is much better when the working frequency is beyond the selected frequency band (transmission coefficients (S21) is less than −30 dB). Second, to map the local field distribution (E_z_) on the graded spoof-plasmonic resonator chain we adopt a microwave near-field imaging technique^27^. Fig. 4a–e show the measured E_z_-field distributions and phase information, at different frequencies, 1 mm above the top of the graded chain. The numerically simulated fields are also shown in Fig. 4f–j for comparison, which are in good agreement with the measurement results. We observe from the measured near-field patterns that localized spoof-SPPs can be cutoff at designed positions for different frequencies due to the graded couplings between neighboring resonators along the graded chain. In general, two different types of electric field distributions can be observed in Fig. 4a–e. Within the waveguiding band of the periodic infinite spoof-plasmonic resonators chain with inter-cavity distance *D* = 10 mm (the last resonator pair), the coupled localized spoof-SPPs can propagate through as an extended mode, as shown in Fig. 4c. However, above or below the waveguiding band of the resonators chain with inter-cavity distance *D* = 10 mm, the coupled localized spoof-SPPs cannot propagate and cutoff at designed positions under different frequencies, as shown in Fig. 4a,b,d,e.

## Discussion

In conclusion, we have experimentally and numerically investigated coupled localized spoof-SPPs in a graded spoof-SPP resonator chain with linearly increasing spacing between neighboring resonators. Because of the gradually varying coupling strength along this special structure, the effective dispersion curves of the graded structure are spatially inhomogeneous. Experimental measurements on this graded chain demonstrate that localized spoof-SPPs can propagate to different positions at different frequencies. This finding may find potential applications in integrated plasmonic circuits.

## Methods

### Sample fabrication

All samples are fabricated using 0.254-mm-thick RT/duroid 5880 (Permittivity ε = 2.2 ± 0.02; Loss tangent Δδ = 0.0009) printed circuit boards with one side covered by 18-μm-thick textured copper disks etched based on the designs.

### Characterization

In the near-field scanning experiment, the source was a 5 mm-long monopole antenna that was placed 0.5 mm away (as indicated by red dots) from the resonator. The probe, another 5 mm-long monopole antenna, was able to scan in the horizontal plane 1 mm above the sample, measuring both the amplitude and phase of the resonance wave fields. By stepping the probe with 0.5 mm increment in both x and y directions, a full 2D spatial field map can be obtained experimentally. Both the souce and probe are connected to a vector network analyzer (R&S®ZVL13).

## Additional Information

**How to cite this article**: Gao, Z. *et al.* Frequency-selective propagation of localized spoof surface plasmons in a graded plasmonic resonator chain. *Sci. Rep.* **6**, 25576; doi: 10.1038/srep25576 (2016).
